# GAPDH Gene Family in *Populus deltoides*: Genome-Wide Identification, Structural Analysis, and Expression Analysis Under Drought Stress

**DOI:** 10.3390/ijms26010335

**Published:** 2025-01-02

**Authors:** Hyemin Lim, Michael Immanuel Jesse Denison, Sathishkumar Natarajan, Kyungmi Lee, Changyoung Oh, Danbe Park

**Affiliations:** 1 Department of Forest Bioresources, National Institute of Forest Science, Suwon 16631, Republic of Korea; kmile@korea.kr (K.L.); happyohcy@korea.kr (C.O.); danbepark@korea.kr (D.P.); 2 3BIGS Company Limited, Hwaseong 18469, Republic of Korea; michael@3bigs.com (M.I.J.D.); sathish@3bigs.com (S.N.)

**Keywords:** genome-wide analysis, *GAPDH* genes, drought stress, gene expression, *Populus deltoides*

## Abstract

Glyceraldehyde-3-phosphate dehydrogenase (GAPDH) is an enzyme widely involved in glycolysis in animal cells and in non-metabolic processes, including apoptosis and the regulation of gene expression. GAPDH is a ubiquitous protein that plays a pivotal role in plant metabolism and handling of stress responses. However, its function in plant stress resistance remains unknown. Identification and systematic analysis of the GAPDH family in *Populus deltoides* (*P. deltoides*) have not been performed. Bioinformatics methods were used to analyze the physicochemical characteristics, structural characteristics, phylogenetic relationships, gene structure, motif analysis, and expression of *GAPDH* gene family members in *P. deltoides*. We identified 12 GAPDH members in *P. deltoides*. Five types of *PdGAPDH* were identified: *GAPA*, *GAPB*, *GAPC1*, *GAPC2*, and *GAPCp*. *PdGAPDH* genes were differentially expressed in leaves, stems, and roots of 1-year-old poplar seedlings. *PdGAPDH* gene transcripts showed that *PdGAPDH2* and *PdGAPDH4* were highly expressed in the leaves. In the roots, seven genes—*PdGAPDH01*, *PdGAPDH05*, *PdGAPDH06*, *PdGAPDH07*, *PdGAPDH08*, *PdGAPDH09*, and *PdGAPDH12*—showed significantly high expression levels. *PdGAPDH02*, *PdGAPDH03*, *PdGAPDH04*, and *PdGAPDH11* showed decreased expression under drought conditions and recovered after re-watering. These results lay the foundation for further studies on the drought stress mechanisms of *P. deltoides.*

## 1. Introduction

Poplar is a model tree species, with approximately 30 species native to the Northern Hemisphere that are fast-growing, highly adaptable, and have excellent characteristics. The genus Populus can be divided into six sections based on leaf and flower shape: Abaso, Aigeiros, Leucoides, Populus, Tacamahaca, and Turanga [1]. *Populus deltoides* is native to the southeastern United States and southern Canada and is taxonomically included in the genus Populus, section Aigeiros [1]. *P. deltoides* has important ecological and economic value and is widely used in poplar breeding programs owing to its excellent characteristics, such as rapid growth, disease resistance, moderate genome size, ease of experimental manipulation, and availability of genome information [2]. *P. deltoides* plays an important role in forest breeding research and has become the main gene donor for poplar cultivars [3].

Globally, the climate crisis has increased exposure to environmental stress factors such as temperature extremes (both low and high), soil drought, and high salinity, which have had a significant impact on plant survival and productivity [4]. Drought is one of the most important abiotic stress factors affecting the natural distribution of woody plants and limits ecosystem production worldwide [4]. Understanding the mechanisms through which plants respond to environmental stress is important for their growth and development. Therefore, it is necessary to identify and explore stress-responsive genes and clarify their regulatory mechanisms.

Glyceraldehyde-3-phosphate dehydrogenase (EC 1.2.1.12) (GAPDH), a key enzyme in the glycolytic pathway, catalyzes the oxidation of glyceraldehyde 3-phosphate (GAP) to 1,3-bisphosphoglycerate (1,3-BPG). Initially, the *GAPDH* gene was considered a useful internal reference gene because it is expressed at a constant level in almost all tissues, even under certain stress conditions [5]. However, subsequent studies have demonstrated that GAPDH is unsuitable as a housekeeping gene [6,7]. Additionally, in plants, GAPDH has functional roles in growth and development, energy metabolism, autophagy, abiotic stress tolerance, and disease resistance. Phosphorylation of GAPDH contributes to its functional activity [8].

With the completion of genome sequencing of the gramineous model plants Arabidopsis and rice, the GAPDH gene family has been extensively identified and characterized in many plants. Arabidopsis contains seven isoforms, including two cytoplasmic members (GAPC1/GAPC2), three chloroplast isoforms (GAPA1/GAPA2/GAPB), and two plastid isoforms (GAPCp1/GAPCp2) [9]. In *Brassica napus*, GAPDH consists of 28 isoforms, of which *BnaGAPDH17*, *BnaGAPDH20*, *BnaGAPDH21*, and *BnaGAPDH22* have been reported to be sensitive to *Sclerotinia sclerotiorum* infection, oxalic acid, and hormone signals [8]. In soybean, overexpression of the *GmGAPDH14* gene exposed to salt stress resulted in a lower content of malondialdehyde (MDA), which suggests enhanced tolerance to salt stress [10]. The wheat GAPDH gene family consists of 22 genes divided into four types, and their expression analysis indicates that GAPDH plays a role in abiotic stress tolerance [11]. Furthermore, overexpression of *TaGApC2* following drought treatment of *Arabidopsis* revealed that *TaGAPC2* interacted with TaPLD, resulting in decreased ROS production, increased root length, and increased drought tolerance [12]. Among 27 upland cotton *GAPDH* gene members, *Gh_GAPDH9* was confirmed to positively regulate drought tolerance [13]. Eight *GAPDH* genes have been identified in watermelon, which are expressed with remarkable tissue specificity and follow diverse expression patterns in response to H_2_O_2_, cold, salt, osmotic stress, heat, and phytohormones [14]. In the sweet orange (*Citrus sinensis*) genome, six *GAPDH* genes have been identified: CsGAPDH1, 2, and 3, which are included in the GAPC group; CsGAPDH4, which is included in the GAPCp group; and CsGAPDH5 and 6, which are included in the GAPA/B group [15]. In addition, the red oak (*Quercus rubra*) genome has 13 *GAPDH* genes, including 1 GAPB chloroplast isoform, 3 GAPA-2 cytoplasmic members, 7 GAPC2, and 2 GAPCP-2 members [16]. However, few reports have described the properties and functions of GAPDH in woody plants. High-quality whole-genome sequencing has made this work possible in *P. deltoides*.

Here, we identified 12 members of the *GAPDH* gene family in *P. deltoides* and analyzed their phylogenetic relationships, chromosomal locations, *cis*-acting elements, gene structures, and collinearity. Additionally, we analyzed *PdGAPDH* gene expression patterns in different tissues under drought stress and re-watering conditions. Systematic analysis of the characterization and evolution of the GAPDH gene family will help to elucidate the regulatory mechanisms of GAPDH and provide an important reference for tree breeding.

## 2. Results

### 2.1. Identification of GAPDH Gene Family Protein and Physicochemical Characterization of PdGAPDH

Overall, 12 GAPDH proteins with conserved Gp_dh_C and Gp_dh_N domains were recognized as members of the *P. deltoides* GAPDH family and named *PdGAPDH01*-*PdGAPDH12* based on their chromosomal locations (Table 1). The physicochemical properties of these PdGAPDH proteins, which varied in length, molecular weight, theoretical isoelectric point, and other properties, were analyzed, as shown in Table 1. The *PdGAPDH* genes were predicted to encode polypeptides of 337 to 463 amino acids, with predicted molecular weights ranging from 36.6 to 49.6 kDa. The theoretical pI ranged from 6.61 to 8.44, the aliphatic index ranged between 81.35 and 93.36, while the grand average of the hydropathicity values of all PdGAPDH proteins was negative, ranging from −0.11 to −0.02, indicating a hydrophilic property. The predicted number of negatively charged residues (Asp + Glu) in PdGAPDH was 41–50, and the number of positively charged residues (Arg + Lys) was 40–52.

### 2.2. Phylogenetic Tree and Subcellular Localization of GAPDH Gene Family

**Figure 1:**
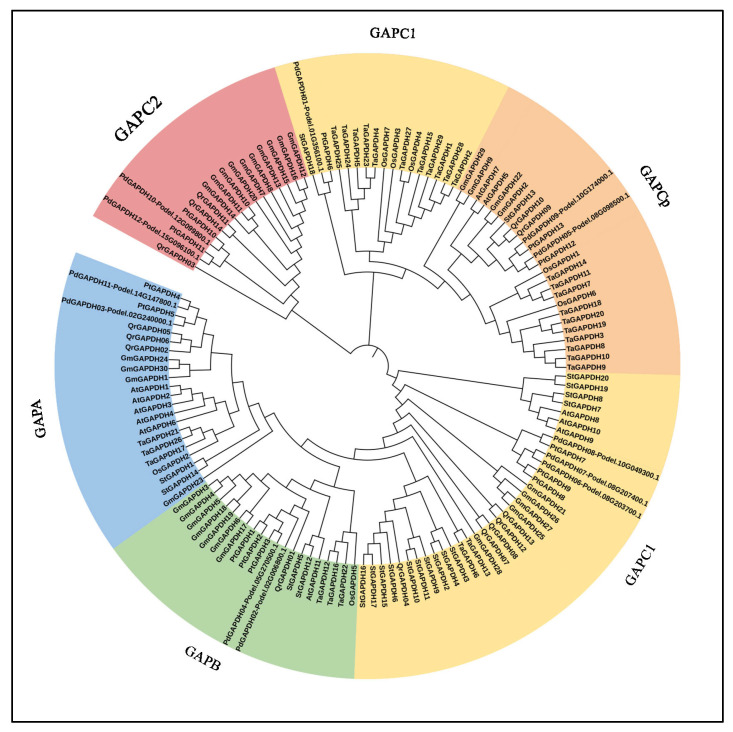
Comparison of the phylogenetic tree of GAPDH in closely related species (At, *Arabidopsis thaliana*; Os, *Oryza sativa*; Ta, *Triticum aestivum*; Gm, *Glycine max*; St, *Solanum tuberosum;* Qr, *Quercus rubra* and *Pd, Populus deltoides*). The placement of the 12 GAPDHs of *P. deltoides* in the circular phylogenetic tree is distinctly shown using database IDs.

To elucidate the evolutionary relationships of the GAPDH proteins, an NJ phylogenetic tree was constructed with 12 PdGAPDHs, and other species, such as *Arabidopsis thaliana*, *Oryza sativa*, *Triticum aestivum*, *Glycine max*, *Solanum tuberosum, Quercus rubra* and *Populus trichocarpa* were analyzed. The constructed phylogenetic tree is shown in Figure 1.

The GAPDH members of *A. thaliana* were denoted as AtGAPDH1–AtGAPDH11 (11 genes), *O. sativa* as OsGAPDH1–OsGAPDH7 (7 genes), *T. aestivum* as TaGAPDH1–TaGAPDH29 (29 genes), and *S. tuberosum* as StGAPDH1–StGAPDH19 (19 genes). The GAPDH family proteins from different plants were identified and extracted using the hmm-based approach with pfams, Pfam00044 and Pfam02800. Only complete genes containing the C-terminal and N-terminal domains were used for comparative analysis. Protein sequences were subjected to a BLASTp search against the nr database of *A. thaliana* to classify the 12 *GAPDH* genes. The *GAPDH* genes in *P. deltoides* were classified into five different types: *GAPA*, *GAPB*, *GAPC1*, *GAPC2*, and *GAPCP* (Table 1).

Podel.08G203700.1, Podel.08G207400.1, and Podel.10G049300.1 proteins clustered in clade I, as highlighted in red (Figure 1). The three proteins in clade I denote GAPC1 and are grouped with other GAPC-1 proteins from other species. Podel.08G098500.1 and Podel.10G174000.1 were located in Clade III (highlighted in green). The two proteins associated with clade III were denoted as GAPCP-2. In Clade IV, we observed the clustering of a few proteins, such as Podel.02G240000.1. p and Podel.14G147800.1 (highlighted in blue), belonging to GAPA-1. Proteins including Podel.02G006800.1. p and Podel.05G270500.1 were found in the clade indicated in yellow; GAPB clustered to clade V. In addition, other proteins such as Podel.01G356100.1, Podel.12G099800.1, and Podel.15G096100.1 belonged to GAPCs.

**Figure 2:**
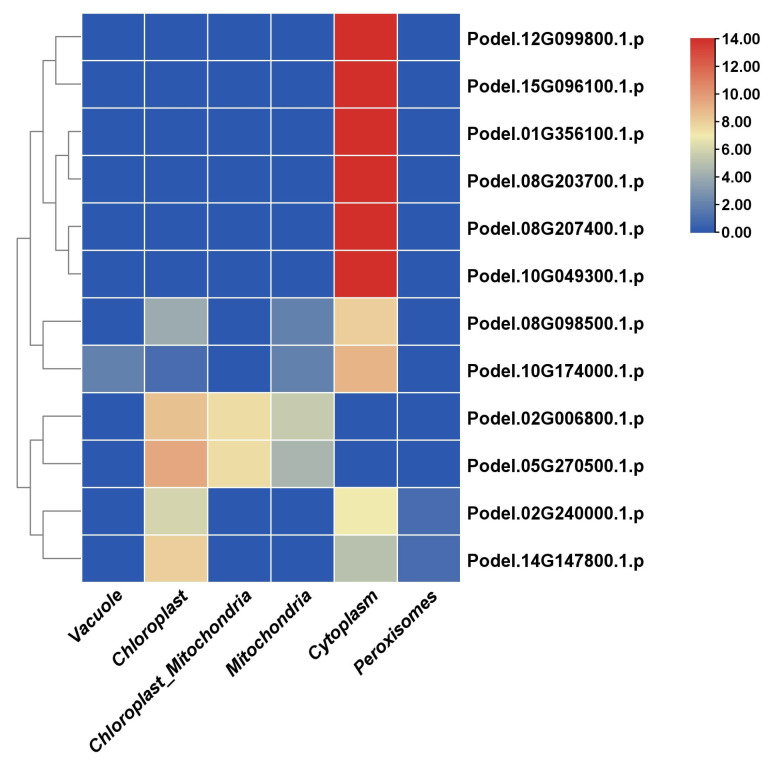
Subcellular localization of PdGAPDH proteins. The color intensity values in the heatmap indicate the number of nearest neighbors to the query calculated using k-nearest neighbors.

Subcellular localization studies using the WOLF PSortB tool revealed that the GAPDHs were mostly localized in the cytoplasm. Specifically, the protein families Podel.12G099800.1, Podel.15G096100.1, Podel.01G356100.1, Podel.08G203700.1, Podel.08G207400.1, and Podel.10G049300.1, were mainly localized in the cytoplasm, whereas a few GAPDHs, such as Podel.08G098500.1, Podel.10G174000.1, Podel.02G240000.1, and Podel.14G147800.1, were also found in the cytoplasm. The proteins Podel.02G240000.1 and Podel.14G147800.1 were also found in low quantities in chloroplasts. Finally, the PdGAPDHs Podel.02G006800.1 and Podel.05G270500.1 were localized in both the chloroplasts and mitochondria (Figure 2).

### 2.3. Cis-Regulatory Element and GO Analysis of the GAPDH Proteins of P. deltoides

**Figure 3:**
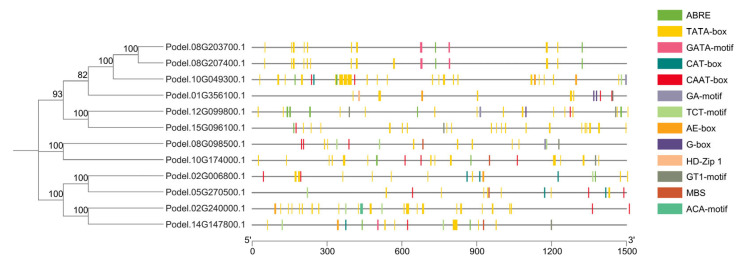
*Cis*-elements in *PdGAPDH* gene promoters.

The *cis*-regulatory elements in each gene type are compared in Figure 3. We observed 244 TATA-box elements across 12 *GADPH* genes. Podel.10G049300.1 (GAPC1) harbors the highest number of TATA-box elements, with 49, followed by the genes Podel.02G240000.1 (GAPA) and Podel.15G096100.1 (GAPC2). Unlike the TATA-box element, very few other elements were identified, including ABRE (20), CAAT box (20), TCT motif (10), CAT box (7), GT1-motif (7), AE box (6), GATA motif (5), MBS (4), GA motif (3), G box (3), ACA-motif (1), and HD-Zip1 (1). GO analysis mapped the proteins encoded by these genes to different biological processes (BP), molecular functions (MF), and cellular structural components (CC). The *GAPDH* genes were mapped to the GO terms: glucose metabolic process, response to sucrose, and the reductive pentose–phosphate cycle under biological process; glyceraldehyde-3-phosphate dehydrogenase (NAD^+^) (phosphorylating) activity, protein homodimerization activity, glyceraldehyde-3-phosphate dehydrogenase (NADP^+^) (phosphorylating) activity, NADP binding, NAD binding, and disordered domain-specific binding under molecular function; chloroplast stroma, stromule, apoplast, and supramolecular complex under cellular component (Table S1).

### 2.4. Chromosomal Location, Gene Duplication, Collinearity, and Synteny Analysis

**Figure 4:**
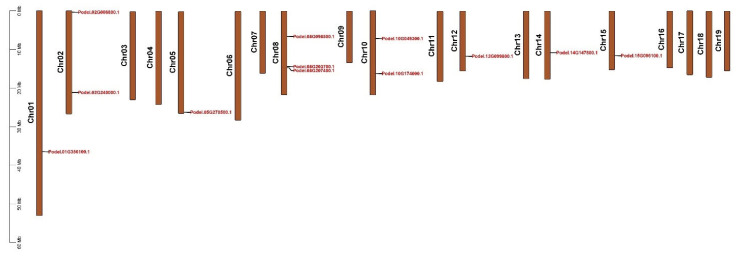
Chromosome location of the *GAPDH* gene family in *P. deltoides*.

PdGAPDHs were unevenly mapped onto 8 of the 19 chromosomes of *P. deltoides*, as shown in Figure 4, based on the annotation information from the *P. deltoides* genome. A maximum of three genes and a minimum of one gene were mapped to several chromosomes. For instance, Podel.08G098500.1, Podel.08G203700.1, and Podel.08G207400.1 were mapped to chromosome 8. Podel.02G006800.1 and Podel.02G240000.1 were mapped to chromosome 2, whereas Podel.10G049300.1 and Podel.10G174000.1 were mapped to chromosome 10. Only one gene was identified on chromosomes 01, 05, 12, 14, and 15. This indicates an even distribution of genes across *P. deltoides* chromosomes.

To explore the genomic expansion mechanism of the *GAPDH* gene family in *P. deltoides*, the syntenic relationships of PdGAPDHs were analyzed. In total, all 12 PdGAPDHs (two gene pairs) had syntenic relationships, and the two gene pairs had undergone tandem duplications (Table 2). The Ka/Ks substitution rate ratio is useful for detecting selective pressures during gene duplication. The Ka/Ks ratios of duplicated pairs with Ka/Ks ratios < 1 indicate genes that may have undergone purifying selection. It is generally believed that while the Ks value is not affected by natural selection, the Ka value is. Overall, the divergence times ranged from 89.58 million years ago (Mya) to 16.89 Mya. The gene pair Podel.01G356100.1 (GAPC1)/Podel.15G096100.1 (GAPC2) underwent whole-genome duplication/segmental event approximately 89.58 Mya. The gene pairs in which whole-genome duplication occurred > 80 Mya denote the evolution of new variants, while other gene pairs of <80 Mya did not evolve new variants.

**Figure 5:**
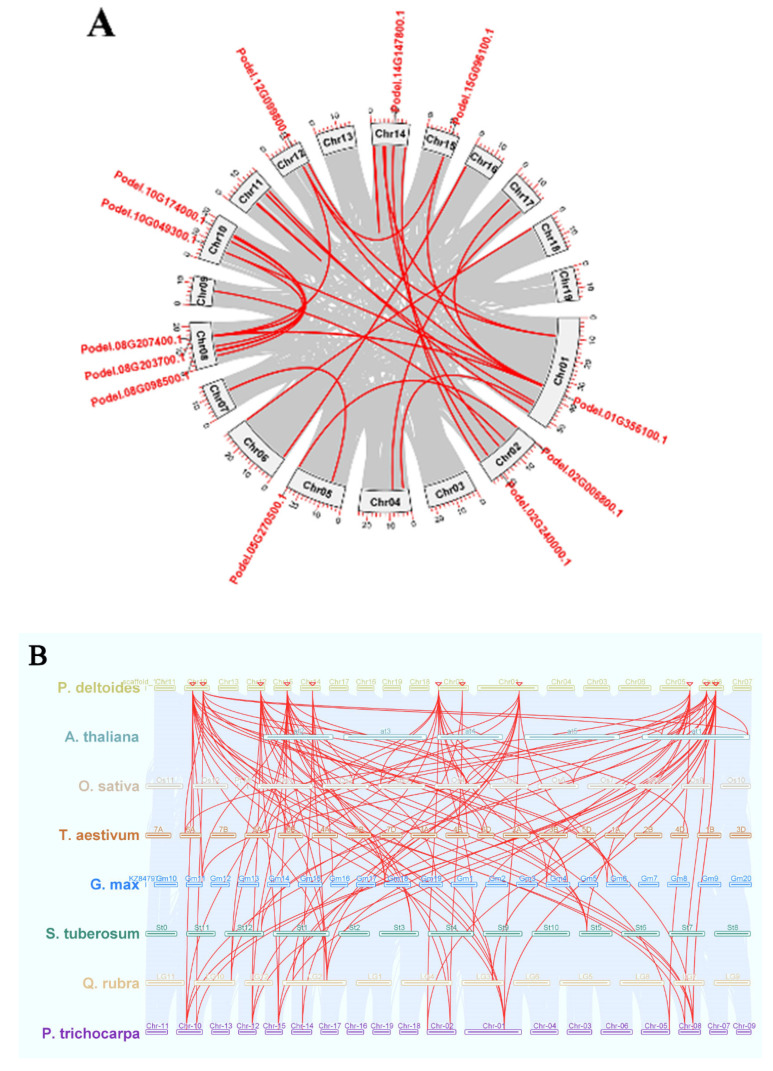
Collinearity and synteny analysis of the *PdGAPDH* gene family. (**A**) Synteny analysis of *PdGAPDH* genes in *P. deltoides*. Chromosomes are represented as LG along with numbering. Red curves indicate syntenic gene pairs. Gray lines indicate syntenic blocks. (**B**) Collinearity analysis of *GAPDH* genes among *A. thaliana*, *O. sativa*, *T. aestivum*, *G. max*, *S. tuberosum*, *Q. rubra,* and *P. trichocarpa* genomes. Gray lines indicate collinear blocks within the six genomes, while red lines represent collinear *GAPDH* gene pairs. The small inverted red triangle (∇) indicates the location of *GAPDH* genes on the chromosomes of *P. deltoides*.

To better understand the genetic divergence, gene duplication, and evolution between the *GAPDH* gene families of *P. deltoides*, Arabidopsis, rice, wheat, soybean, potato, oak trees, and poplar trees, syntenic relationships were analyzed using MCScanX to identify orthologous *GAPDH* genes between these species. Overall, 4, 11, 14, 19, 11, and 17 pairs of orthologous *GAPDH* genes were detected in five comparisons (*P. deltoides* vs. *A. thaliana*, *P. deltoides* vs. *O. sativa*, *P. deltoides* vs. *T. aestivum*, *P. deltoides* vs. *G. max*, *P. deltoides* vs. *S. tuberosum*, *P. deltoides* vs. *Q. rubra,* and *P. deltoides* vs. *P. trichocarpa*) (Figure 5). Collinearity analysis against the previously reported species of poplar tree, *P. trichocarpa*, identified two putative genes, *Potri.002G007100.2* and *Potri.008G179300.1*, while other genes in *P. deltoides* were collinear with those of *P. trichocarpa*.

### 2.5. Gene Structure and Conserved Motifs Analysis of GAPDH Genes

**Figure 6:**
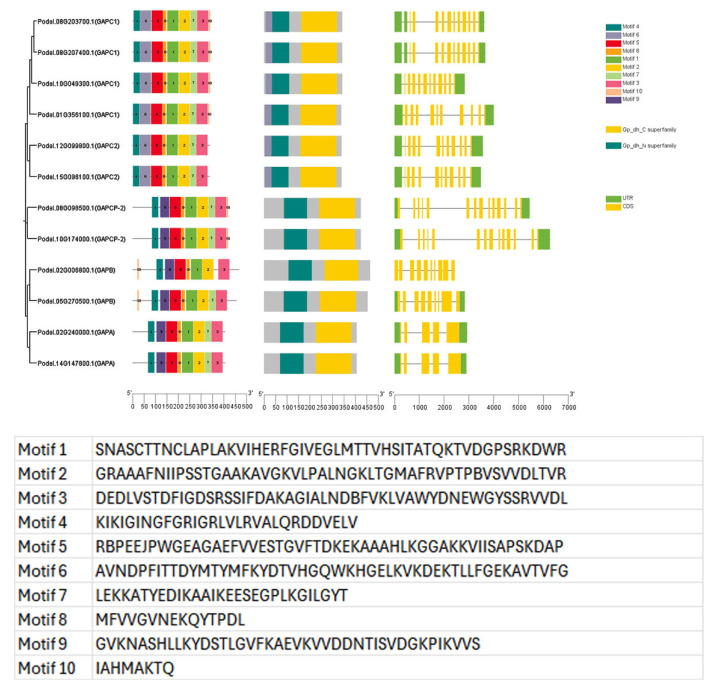
Exon/intron structure and phylogenetic tree based on conserved motifs.

To gain insight into the sequence characteristics of PdGAPDH proteins in *P. deltoides*, we performed motif analysis using MEME, which identified 10 conserved motifs (Figure 6). PdGAPDH proteins within the same phylogenetic group shared a similar motif composition and arrangement. The phylogenetic analysis indicated that there were two main clades: one main clade was occupied by both GAPC1 and GAPC2 with motif 6, whereas the other clade with motif 9 was occupied by both GAPA and GAPB. We also observed that motif 10 was present only in the GAPB-, GAPC1-, and GAPCP-2-type genes. Major differences in motif analysis are known to affect protein topology.

The domain information and exon/intron structures of the 12 *PdGAPDH* genes and coding sequence (CDS)/intron patterns were visualized based on the CDS and genomic sequences. The exon/intron structure differed across *PdGAPDH* genes; however, the Gp_dh_N and Gp_dh_C domains were conserved. The structural arrangement and number of exons and introns were concordant with the phylogenetic placement of the *PdGAPDH* genes. Consequently, most PdGAPDH genes in the same subgroup exhibited similar motif compositions, gene structures, and conserved domain distributions, which further validated the evolutionary relationship analysis results.

### 2.6. Three-Dimensional Structure Modeling

**Figure 7:**
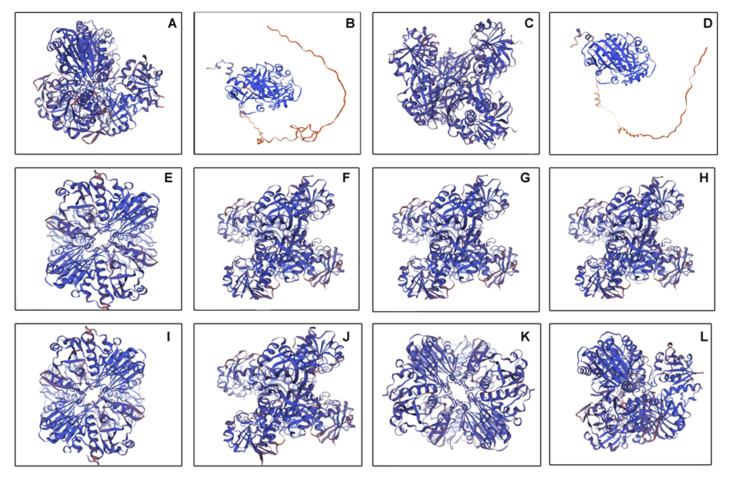
PdGAPDH 3D protein structure. PdGAPDH topology was predicted by homology modeling using Swiss-Model and shown as cartoons using PyMOL. The model is depicted with a color gradient that represents its local quality, ranging from blue (indicating high quality) to red (indicating low quality), based on the all-atom IDDT score. (**A**) Podel.01G356100.1, (**B**) Podel.02G006800.1, (**C**) Podel.02G240000.1, (**D**) Podel.05G270500.1, (**E**) Podel.08G098500.1, (**F**) Podel.08G203700.1, (**G**) Podel.08G207400.1, (**H**) Podel.10G049300.1, (**I**) Podel.10G174000.1, (**J**) Podel.12G099800.1, (**K**) Podel.14G147800.1 and (**L**) Lodel.15G096100.1.

To further characterize the new features identified in the PdGAPDH proteins, three-dimensional (3D) structures of some PdGAPDH proteins were obtained by homology modeling using the Swiss-Model in silico tool. Two GAPA proteins, two GAPB proteins, two GAPCP-2 proteins, four GAPC1 proteins, and four GAPC2 proteins were modeled. Each protein category exhibited a similar topological structure (Figure 7). The major template used for homology-based modeling of PdGAPDH belongs to the PDB ID: 3E5R, the crystal structure and functional analysis of GAPDH from *O. Sativa.*

### 2.7. Protein–Protein Interaction Network Analysis

**Figure 8:**
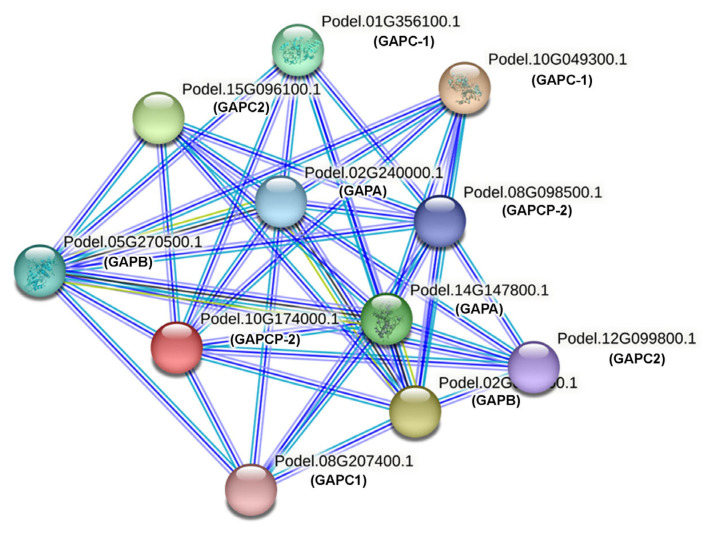
STRING Protein–protein interaction network illustrates the 12 GAPDH proteins identified in *P. deltoides*. In this network, the nodes, represented as colored circles, correspond to proteins, with each node encapsulating all proteins generated by a single protein-coding gene, including various splicing members and alternative polyadenylation forms. The edges, depicted as colored lines connecting the nodes, signify the different types of interactions identified: gene fusions are represented by red lines, gene neighborhoods by green lines, co-occurrences across species by blue lines, experimental evidence by purple lines, text mining from literature abstracts by yellow lines, database interactions by light blue lines, and co-expression within the same or different species.

To better understand the results of protein–protein interaction network results, three different clusters are presented (Figure 8, Table S2). In cluster I, five protein members interacted with each other, including Podel.01G356100.1, Podel.10G049300.1, Podel.10G174000.1, Podel.12G099800.1, and Podel.15G096100.1. In cluster 2, four members interacted with each other, including the protein members Podel.02G006800.1, Podel.02G240000.1, Podel.05G270500.1, and Podel.14G147800.1. The protein members Podel.08G098500.1 and Podel.08G207400.1 interacted with each other.

### 2.8. Expression Patterns of PdGAPDH Genes Under Drought Stress and Re-Watering

**Figure 9:**
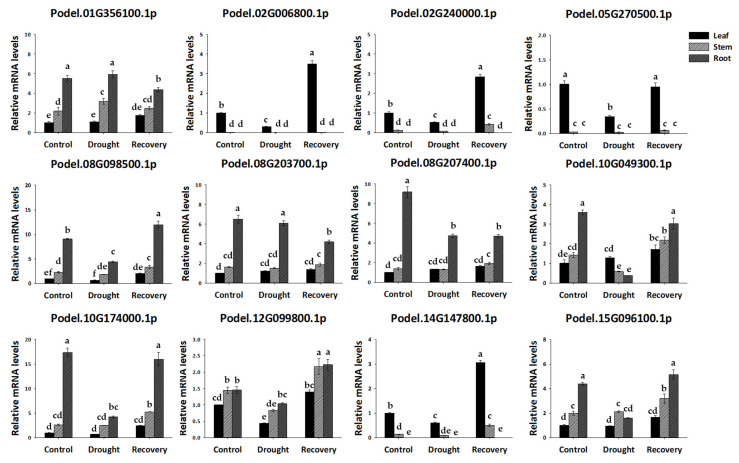
Relative expression of 12 *PdGAPDH* genes under drought stress and in different tissues. The standard deviation determined from three biological replicates is shown with error bars. Significant differences (one-way ANOVA, *p* < 0.05) are indicated by different lowercase letters.

Using qPCR analysis, the transcript levels of the *PdGAPDH* gene were determined in leaves, stems, and roots. The expression of the *PdGAPDH* gene showed different patterns depending on the tissue type (Figure 9). In particular, the chloroplast genes PdGAPDH2 (Podel.02G006800.1) and PdGAPDH4 (Podel.05G270500.1) were prominently expressed in the leaves (Figure 9). PdGAPDH3 (Podel.02G240000.1) and PdGAPDH11 (Podel.14G147800.1) genes, which are GAPAs and belong to group IV, showed significantly higher expression in the leaf and stem tissues. The genes that showed the highest levels of expression in the roots were identified as PdGAPDH1 (Podel.01G356100.1), PdGAPDH5 (Podel.08G098500.1), PdGAPDH6 (Podel.08G203700.1), PdGAPDH7 (Podel.08G207400.1), PdGAPDH8 (Podel.10G049300.1), PdGAPDH9 (Podel.10G174000.1), and PdGAPDH12 (Podel.15G096100.1).

Based on the analysis of *cis*-acting elements located 1500 bp upstream of *PdGAPDH*, we confirmed that the promoter region of *PdGAPDH* contained regulatory elements that were responsive to drought stress. Changes in the expression of *PdGAPDH* in *P. deltoides* (Ay48 clone) under the influence of drought stress were analyzed. As shown in Figure 9, qPCR analysis indicated that the relative expression of *PdGAPDH02*, *PdGAPDH03*, *PdGAPDH04*, and *PdGAPDH11* in leaf tissues was significantly lower than those in the control group after 12 days of drought treatment and recovered after 4 days of re-watering. In addition, the relative expression of *PdGAPDH05*, *PdGAPDH08*, *PdGAPDH09*, and *PdGAPDH12* in the root tissues was significantly lower after drought treatment and recovered after re-watering.

## 3. Discussion

Forestry and agricultural productivity are expected to decline significantly because of both biotic and abiotic pressures, which pose a substantial threat to the security and equilibrium of the biosphere and global energy supply. To maintain this balance, plants require effective regulatory systems that harmonize environmental and developmental inputs across various tissues [17]. Additionally, GAPDH facilitates a range of precisely regulated modifications to sensitive cysteine residues, enabling plants to adapt to diverse external stressors of varying intensity and nature [18]. The functional role of GAPDH, a crucial enzyme in the glycolysis metabolic pathway, has underscored its importance in energy metabolism, plant growth, and development [19]. Despite extensive functional studies on GAPDH enzymes in model organisms, such as rice and Arabidopsis, research focusing on the characterization and functions of GAPDH in poplar species remains limited. Given the critical role of GAPDH enzymes, a comprehensive investigation of the evolutionary and functional aspects of the *GAPDH* gene family is essential for understanding their regulatory mechanisms and offers valuable insights for genetic engineering in breeding programs [8].

### 3.1. Physicochemical Characteristics of PdGAPDH Protein

The isoelectric point (pI), along with the balance between positively and negatively charged residues, plays a crucial role in determining solubility, interactions, and subcellular localization. The pI refers to the pH level at which proteins exhibit neutrality and is characterized by an equal distribution of positive and negative ions. Among the GAPDH proteins studied in *P. deltoides*, eight exhibited pI values exceeding seven, whereas two GAPDH proteins from Populus were identified as neutral. Notably, only two GAPDH members were found to be acidic [20]. The instability index (II) serves as an indicator of protein stability both in vitro and in vivo. Proteins were deemed stable if their instability index (II) was <40, whereas those with an index of >40 were classified as unstable [21]. The stability index (II) of GAPDH was <40, indicating that these proteins were stable. Another important measure for evaluating protein stability is the aliphatic index (AI). This index represents the volume fraction occupied by the aliphatic side chains of amino acids, including alanine (A), valine (V), leucine (L), and isoleucine (I). This study investigated the correlation between thermostability and aliphatic indices [22], where a high AI value indicated that the protein maintained its stability across a wide temperature spectrum. The stability of GAPDHs in *P. deltoides* was further supported by their higher aliphatic index compared to that of other GAPDH proteins, as evidenced by Podel.05G270500.1 and Podel.14G147800.1.1. In addition to evaluating the protein stability and concentration, the GRAVY score served as a measure of the hydrophobic or hydrophilic characteristics of a protein. This score was calculated by dividing the total hydropathy values of all the amino acids within the protein by the total number of residues present. As established by [23], the GRAVY score can range from −2 to +2, with negative values indicating hydrophilicity and positive values indicating hydrophobicity. Conversely, proteins with higher GRAVY scores are generally more hydrophilic and show enhanced solubility. A protein with a GRAVY score exceeding 0.4 may be hydrophobic, making it difficult to visualize on 2-D gels, as noted by [24].

### 3.2. Cis-Regulatory Elements

Multifunctional proteins such as GAPDH display unique functions that are not associated with their initial roles. In addition to glycolysis, GAPDH is involved in the maintenance of DNA integrity, histone production, iron metabolism, membrane trafficking, and receptor-mediated cell signaling. Furthermore, the intracellular locations of multifunctional proteins vary significantly, which reflects their changing functions. GAPDH is a cytosolic protein found in the cell membrane, nucleus, polysomes, endoplasmic reticulum, and Golgi apparatus [18]. Our study showed that a limited number of GAPDHs expressed in *P. deltoides* were identified in the chloroplasts and mitochondria, whereas most of the investigated GAPDH proteins appeared to be located in the cytoplasm. The GAPDHs that reside in the cytoplasm are known as GAPCs, whereas those in the chloroplasts and mitochondria are known as GAPA and GAPB. GAPDHs have long been recognized as enzymes involved in energy metabolism and the production of ATP and pyruvate through anaerobic glycolysis in the cytoplasm. The *cis*-regulatory elements (CREs) encode genomic blueprints that ensure proper spatiotemporal patterning of gene expression necessary for appropriate development and responses to the environment. The most widely studied CREs in plants include the abscisic acid (ABA)-responsive element (ABRE) and dehydration-responsive element (DRE)/CRT (C-repeat) elements, which are largely implicated in stress responses. Additionally, the CArG-box, AuxRE, and E2F-binding elements are involved in plant development, while MBS elements are implicated in myriad developmental processes and stress responses [25]. In this study, we found that CREs, such as ABREs and MBS, were significantly detected in the promoter regions of GAPDH members, which could assist plants under stress conditions. In addition, the majority of TATA-box and CAAT-box genes were present in abundance in all 12 *GAPDH* genes. Genome-wide studies of the *DREB* gene family in tomato during drought and heat responses showed that TATA-box and CAAT-box elements were found in larger numbers, while ABRE and MBS motifs were also detected [26]. Similar results were observed in a study involving *cis*-regulatory elements that control the regulation of the *NHX* gene family in potato [27].

### 3.3. Collinearity and Syntenic Relationship with Other Crops’ Genomes

Chromosomal allocation studies have implied that *GAPDH* gene expansion occurs because of segmental duplication. In *P. deltoides,* the GAPC1 was duplicated in the time range between 16.88 Mya and 61.27 Mya, whereas GAPC2 was duplicated between 85.89 Mya and 89.58 Mya. The duplication time of GAPC2 was earlier than that of GAPC1, which may indicate a significant role in the expansion of the *GAPDH* gene family. According to Wei et al., 13 PtGAPDHs were identified in *P. trichocarpa*. Among these 13 GAPDHs, only 11 genes were collinear with *P. deltoides PdGAPDH* genes. The non-collinear putative genes in *P. trichocarpa* indicate that those genes might have evolved due to gene duplication among these species [28].

### 3.4. Three-Dimensional Structure Modeling and Protein–Protein Interaction Studies

Three-dimensional structural analysis of GAPDHs suggested that all the GAPC members exhibited identical topological structures, whereas the structure of GAPA appeared to be similar to that of GAPCs. Interestingly, the three-dimensional structure of GAPB was completely different from that of GAPA and GAPCs. GAPA regulates glycolysis, whereas GAPB regulates gluconeogenesis. GAPA/GAPB are present in chloroplasts and require NADP+ as a coenzyme to reduce 1,3-BPG to G3P [29]. We observed different variants of the GAPC proteins, including GAPC1, GAPC2, and GAPCP2. GAPCPs are present in the cytoplasm and plastids, and use NAD+ as a coenzyme. In strawberries, four variants of GAPCs have been detected [29]. In Arabidopsis, the main role of GAPCPs has been identified and includes primary root and microspore development [30].

### 3.5. PdGAPDH Expression Under Drought Stress and Re-Watering

Drought stress significantly affects plant growth, development, and secondary metabolite synthesis [31]. *PdGAPDHs* under drought stress and re-watering revealed three significant expression patterns in the qPCR analysis. The expression of a few GAPDHs, such as Podel.02G006800.1 (GAPB), Podel.02G240000.1 (GAPA), Podel.14G147800.1 (GAPA), and Podel.05G270500.1 (GAPB), was significantly higher in the leaves of the recovered plants. This indicates that GAPDH proteins are expressed in the chloroplasts of *P. deltoides* leaves. GAPDHs such as Podel.08G098500.1 (GAPCP-2) and Podel.10G174000.1 (GAPCP-2) were highly expressed in the roots, whereas no significant expression was observed in other tissues such as leaves and stems. GAPCP-2 is a cytosolic/plastid-localized GAPDH. Other GAPDHs among GAPC1, including Podel.10G049300.1, Podel.12G099800.1, and Podel.15G096100.1, which belong to the GAPC1/2 family, were significantly expressed in all tissues of *P. deltoides* (leaves, stems, and roots). In particular, no genes with unchanged expression levels in the tissues or stress treatments were identified in *P. deltoides*. These results suggest that it would be difficult to use the *PdGAPDH* gene as an internal control.

## 4. Materials and Methods

### 4.1. Identification of the GAPDH Family Genes in P. deltoides and Retrieval of Closely Related Plant Species

The complete protein sequence of *P. deltoides* was downloaded from the Phytozome database (https://phytozome-next.jgi.doe.gov/, accessed on 8 May 2024). The N-terminal Gp_dh_N domain (PF00044) and C-terminal Gp_dh_C domain (PF02800) are the primary features of GAPDH protein monomers, which are highly conserved in all living organisms. All of the resulting GAPDH proteins were submitted to a Batch-CD search (https://www.ncbi.nlm.nih.gov/Structure/bwrpsb/bwrpsb.cgi, accessed on 8 May 2024) to confirm the conserved GAPDH N- and C-terminal domains, and those proteins containing both the N- and C-terminal domains were selected for further analysis. Proteins lacking either terminal domain were discarded. Transcripts with lower e-values in the HMM model were selected as *GAPDH*. The *GAPDH* gene in *P. deltoides* was designated as *PdGAPDH*. We also downloaded the assemblies and GFF files from closely related species, such as *Arabidopsis thaliana*, *Oryza sativa*, *Triticum aestivum*, *Glycine max*, *Solanum tuberosum,* and *Quercus rubra*. The GAPDHs of *O. sativa*, *T. aestivum*, *G. max*, *S. tuberosum,* and *Q. rubra* can be abbreviated as *OsGAPDH*, *TaGAPDH*, *GmGAPDH*, *StGAPDH,* and *QrGAPDH*, respectively.

### 4.2. Characteristic Analysis of PdGAPDH Protein

The fundamental properties of PdGAPDH, including protein length (Len), molecular weight (MW), isoelectric point (pI), and total hydrophilicity (GRAVY), were examined using the ProtParam web application (https://web.expasy.org/protparam/, viewed on 10 May 2024). The subcellular localization of PdGAPDH proteins was predicted using WoLF PSORTB [32]. The SWISS-MODEL online server (https://www.swissmodel.expasy.org/, accessed 10 May 2024) was used to build the three-dimensional protein model.

### 4.3. Phylogenetic Analysis of P. deltoides with Closely Related Species

Phylogenetic analysis was performed using PdGAPDH protein sequences from *Arabidopsis thaliana*, *Oryza sativa*, *Triticum aestivum*, *Glycine max*, *Solanum tuberosum*, and *Quercus rubra*. Multiple sequence alignments were performed using the default parameters of ClustalW version 2.0 [33]. With 1000 bootstrap replications, an unrooted neighbor-joining (NJ) tree was built using MEGA X [34] software.

### 4.4. Identification of Cis-Regulatory Elements and GO Analysis of the GAPDH Proteins of P. deltoides

The *cis*-acting elements were predicted using the PlantCARE program (http://bioinformatics.psb.ugent.be/webtools/plantcare/html/, accessed on 10 May 2024) using PdGAPDH promoter sequences, which were 1.5 kb upstream of the start codon [35]. TBtools II and Excel were used to perform statistical analyses and visualize the data. The *GADPH* genes were first aligned with the Arabidopsis protein database using local BLASTx, with an E-value of 10^−5^. Based on this annotation, the BLAST2GO program [36] was used to determine the gene ontology (GO) annotation.

### 4.5. Phylogenetic Relationships, Gene Structure and Conserved Motif Analysis of the PdGAPDH Gene Family 

MEGA X was used to carry out domain alignments, which were then input into iToL (https://itol.embl.de/, accessed on 9 May 2024) for visualization [34,37]. Multiple sequence alignments of the GAPDH proteins were performed using ClustalW. A phylogenetic tree with pairwise deletions and 1000 bp replications was constructed using the neighbor-joining (NJ) method. Using genomic sequence and structural annotation data, as well as the Gene Structure Display Server 2.0 (https://gsds.gao-lab.org/Gsds_help.php, accessed on 9 May 2024), the exon/intron structures of the *GAPDH* genes were obtained [38]. The conserved motifs of GAPDH proteins were examined using MEME (http://meme-suite.org/, accessed on 10 May 2024), with a maximum recognition motif of ten [39,40].

### 4.6. Chromosomal Distribution, Collinearity, and Syntenic Analysis of the PdGAPDHs

The positional and structural gene information of *P. deltoides* was downloaded in a Generic Feature Format Version 3 (GFF3) file from Phytozome v13 (https://phytozome-next.jgi.doe.gov/info/PdeltoidesWV94_v2_1, accessed on 7 May 2024) [41]. Next, the *GAPDH* genes were mapped onto the appropriate chromosomes using TBTools (http://mg2c.iask.in/mg2c_v2.0/, accessed on 9 May 2024). The Multiple Collinearity Scan Toolkit (MCScanX) and TBtools [42,43] were used to investigate and report the syntenic relationships of the *GAPDH* genes in *A. thaliana*, *O. sativa*, *T. aestivum*, *G. max*, *S. tuberosum*, and *Q. rubra*. The Ka and Ks values of the segmentally and tandemly duplicated gene pairs were determined using the downstream analysis function of MCScanX. The Ks values were used to determine the time points of duplication occurrence (T) according to the following formula: according to Zhao and co-workers [44], T = Ks/2λ, where λ = 1.5 × 10^−8^ s for dicots. The Ka/Ks ratio was utilized to assess the mode of selection acting on GAPDHs [45]. We examined the NCBI (https://www.ncbi.nlm.nih.gov/, accessed on 6 May 2024) for the functions of the GAPDHs in other species, which we hypothesized to be orthologous genes of the PdGAPDHs. To further investigate the relationships between PdGAPDH proteins, a protein–protein interaction (PPI) network was predicted using orthologous proteins of PdGAPDHs in Arabidopsis. STRING (https://string-db.org/, accessed on 9 May 2024) was used to construct the functional interaction network of the proteins [46].

### 4.7. Plant Materials and Drought Stress Treatment

One-year-old *P. deltoides* (Ay48 clone) were grown in a mixture of topsoil and sand (3:1) in pots with adequate soil moisture in a greenhouse. The plants were watered to a soil moisture level of 40% one day before drought treatment. The plants were then withheld from water for 12 days and observed during this period, followed by a re-watering phase lasting for 4 days. Soil moisture was measured every alternate day using a moisture probe (ICT International Pty. Ltd., Armidale, NSW, Australia). The experiment was conducted under semi-controlled conditions at the National Institute of Forest Science, Suwon, Republic of Korea (37°15′04″ N, 136°57′59″ E). Samples were immediately frozen in liquid nitrogen upon collection and stored at −80 °C until analysis. Each treatment group comprised three replicates.

### 4.8. RNA Extraction and Quantitative Real-Time PCR (qPCR) Assay

For qPCR analysis, total RNAs were isolated from *P. deltoides* seedlings using the Beniprep^®^ Super Plant RNA extraction kit (InVirusTech Co., Gwangju, Republic of Korea), according to the manufacturer’s protocol. RNA samples were then converted into single-stranded cDNA using the cDNA EcoDry Premix (TaKaRa, Shiga, Japan). qPCR was conducted on a CFX96 Touch Real-Time PCR Detection System (Bio-Rad, Hercules, CA, USA) using the IQtm SYBR Green Supermix (Bio-Rad). The reaction conditions were as follows: an initial denaturation at 95 °C for 30 s, followed by 38 cycles of 95 °C for 5 s and 60 °C for 34 s. The relative abundance of transcripts was analyzed using the 2^−ΔΔCt^ method [47]. The expression levels of *Actin* and *UBQ7* were used as housekeeping genes [48]. The gene-specific primers used are listed in Table S3.

## 5. Conclusions

In the present study, we identified 12 *GAPDH* genes in *P. deltoides*. Phylogenetic analysis, physicochemical characterization, gene structure examination, identification of cis-acting regulatory elements, three-dimensional structural analysis, and gene duplication investigations revealed five distinct variants: *GAPA, GAPB, GAPC1/2*, and *GAPCP-2*. Furthermore, phylogenetic analysis revealed that the *GAPDH* gene family can be classified into variants. Collinearity analysis suggested that the genome experienced either whole-genome or segmental duplication, contributing to the expansion of the *GAPDH* gene family in *P. deltoides*. Additionally, the identified *cis*-acting elements implied that these *GAPDH* genes play a role in stress management under drought conditions. The findings of this study provide a foundation for further exploration of the biological functions of the *GAPDH* family genes in *P. deltoides*.

## Figures and Tables

**Table 1:** Gene IDs and physicochemical properties of 12 GAPDH-encoding genes in *P. deltoides*.

Gene ID	Symbol	Database ID	Number of AAs ^z^	MW ^y^ (KDa)	Theoretical pI ^x^	Total Number of Negatively Charged Residues (Asp + Glu)	Total Number of Positively Charged Residues (Arg + Lys)	Instability Index	Aliphatic Index	GRAVY ^w^
PdGAPDH1	*GAPC1*	Podel.01G356100.1	337	36,579.02	7.02	41	41	24.72	90.53	−0.054
PdGAPDH2	*GAPB*	Podel.02G006800.1	463	49,572.67	7.59	49	50	27.71	88.64	−0.042
PdGAPDH3	*GAPA*	Podel.02G240000.1	404	43,204.38	8.44	44	47	23.5	90.94	−0.023
PdGAPDH4	*GAPB*	Podel.05G270500.1	452	48,181.19	8.02	50	52	25.58	93.36	−0.021
PdGAPDH5	*GAPCP-2*	Podel.08G098500.1	422	45,156.33	6.67	46	45	38.72	81.35	−0.086
PdGAPDH6	*GAPC1*	Podel.08G203700.1	341	37,040.42	7.72	43	44	24.05	89.5	−0.088
PdGAPDH7	*GAPC1*	Podel.08G207400.1	341	37,040.42	7.72	43	44	24.05	89.5	−0.088
PdGAPDH8	*GAPC1*	Podel.10G049300.1	341	37,081.55	7.67	42	43	24.61	89.21	−0.086
PdGAPDH9	*GAPCP-2*	Podel.10G174000.1	422	45,018.24	8.13	44	46	35.89	82.27	−0.088
PdGAPDH10	*GAPC2*	Podel.12G099800.1	338	36,739.99	6.61	41	40	22.28	88.82	−0.110
PdGAPDH11	*GAPA*	Podel.14G147800.1	404	43,005.99	8.14	44	46	23.07	92.4	−0.020
PdGAPDH12	*GAPC2*	Podel.15G096100.1	338	36,784.16	7.01	41	41	25.67	87.96	−0.099

^z^ AA, amino acid; ^y^ MW, molecular weight; ^x^ pI, isoelectric point; ^w^ GRAVY, grand average of hydropathy.

**Table 2:** Ka/Ks ratios of duplication for *PdGAPDHs*.

Gene 1	Gene 2	Ka	Ks	Ka_Ks	Divergence Time (Mya ^z^)	Duplication Type
*Podel.01G356100.1* *(GAPC1)*	*Podel.15G096100.1* *(GAPC2)*	0.077188301	1.17529841	0.065675492	89.58067146	WGD or Segmental
*Podel.08G207400.1* *(GAPC1)*	*Podel.12G099800.1* *(GAPC2)*	0.103620702	1.174778436	0.088204464	89.54103933	WGD or Segmental
*Podel.01G356100.1* *(GAPC1)*	*Podel.12G099800.1* *(GAPC2)*	0.0699829	1.126863463	0.062104152	85.88898344	WGD or Segmental
*Podel.01G356100.1* *(GAPC1)*	*Podel.10G049300.1* *(GAPC1)*	0.054241837	0.80391583	0.067472034	61.27407238	WGD or Segmental
*Podel.01G356100.1* *(GAPC1)*	*Podel.08G203700.1* *(GAPC1)*	0.069621675	0.732038305	0.095106601	55.79560252	WGD or Segmental
*Podel.01G356100.1* *(GAPC1)*	*Podel.08G207400.1* *(GAPC1)*	0.069621675	0.732038305	0.095106601	55.79560252	WGD or Segmental
*Podel.08G203700.1* *(GAPC1)*	*Podel.10G049300.1* *(GAPC1)*	0.033867269	0.276494684	0.122487957	21.0742899	WGD or Segmental
*Podel.08G207400.1* *(GAPC1)*	*Podel.10G049300.1* *(GAPC1)*	0.033867269	0.276494684	0.122487957	21.0742899	WGD or Segmental
*Podel.12G099800.1* *(GAPC2)*	*Podel.15G096100.1* *(GAPC2)*	0.026955898	0.270828018	0.099531423	20.64237941	WGD or Segmental
*Podel.02G006800.1* *(GAPB)*	*Podel.05G270500.1* *(GAPB)*	0.013587556	0.248883734	0.05459399	18.96979682	WGD or Segmental
*Podel.02G240000.1* *(GAPA)*	*Podel.14G147800.1* *(GAPA)*	0.018788973	0.245791425	0.076442752	18.73410253	WGD or Segmental
*Podel.08G098500.1* *(GAPCP-2)*	*Podel.10G174000.1* *(GAPCP-2)*	0.030885542	0.221541013	0.139412302	16.88574792	WGD or Segmental

^z^ Mya, million years ago.

## Data Availability

Data is contained within the article and Supplementary Materials.

## Supplementary Materials

The following supporting information can be downloaded at https://www.mdpi.com/article/10.3390/ijms26010335/s1.
